# Psycho-social and health predictors of loneliness in older primary care patients and mediating mechanisms linking comorbidities and loneliness

**DOI:** 10.1186/s12877-023-04436-6

**Published:** 2023-12-04

**Authors:** Ljiljana Majnarić Trtica, Mile Volarić, Tomislav Kurevija, Silvio Mihaljević, Zdravka Krivdić Dupan, Thomas Wittlinger

**Affiliations:** 1 Department of Family Medicine, Faculty of Medicine, Josip Juraj Strossmayer University of Osijek, Huttlerova 4, 31000 Osijek, Croatia; 2 Faculty of Medicine, Josip Juraj Strossmayer University of Osijek, Huttlerova 4, 31000 Osijek, Croatia; 3 School of Medicine, University of Mostar, University Hospital Mostar, Mostar, Bosnia and Herzegovina; 4 Department of Internal Medicine and the History of Medicine, Faculty of Medicine, Josip Juraj Strossmayer University of Osijek, Huttlerova 4, 31000 Osijek, Croatia; 5 Department of Radiology, Faculty of Medicine, Josip Juraj Strossmayer University of Osijek, Huttlerova 4, 31000 Osijek, Croatia; 6 Department of Cardiology, Asklepios Hospital, 38642 Goslar, Germany

**Keywords:** Aging, Loneliness, Psychological factors, Comorbidities, Integrated research approach, Interventions

## Abstract

**Background:**

Aging is associated with many personal, social, and environmental challenges that increase the risk of loneliness. Loneliness is a painful emotional experience associated with a perceived lack of connection and intimacy. Loneliness accelerates health deterioration, but the presence of chronic health conditions (comorbidities) in older individuals may potentiate the feeling of loneliness. The relationships between health status and loneliness in older individuals have not been assessed in an integrated manner, although it is necessary for planning efficient interventions. The aim of this study was to fill in this knowledge gap, by attempting to create an integrated model of loneliness in older individuals.

**Methods:**

The sample consisted of 189 (58% F) older individuals (> 60 years) (mean ± SD, 78.47 ± 6.65), attendees in Primary Health Care. Different factors associated with loneliness in the older population were assessed, and classified as demographic, environmental, physical (health-related), and psychological, in addition to functional abilities. A set of standard questionnaires was used to assess psychological factors and functional abilities. The hierarchical regression model assessed the effect of particular blocks of factors on status loneliness. The second aim was to analyze how psychological factors mediate associations between health status (comorbidity level) and loneliness.

**Results:**

Indicated that increasing comorbidity, anxiety, lack of positive moods, not having hobbies/activities, low perception of social support, impaired cognitive function, and suppression of emotion expression, are significant predictors of loneliness. Mediation analysis informed us of how to help patients with comorbidities feel less lonely. Interventions that were suggested were those that can reduce anxiety and depression, improve cognitive abilities and emotional regulation control, and enhance social support.

**Conclusions:**

Results can help understand the pathophysiology loops linking poor health status (comorbidity level) of older individuals and loneliness, and have significant potentials from the translational perspectives, as a decision-support tool.

**Supplementary Information:**

The online version contains supplementary material available at 10.1186/s12877-023-04436-6.

## Background

Aging is associated with many personal, social, and environmental challenges that may increase the feeling of loneliness [1]. Loneliness is a painful experience accompanying perception that one`s social needs are not satisfied with the actual social relationships [2]. In the core of this feeling is hypervigilance for threat of becoming isolated, which alters one`s behaviors and psychological and physiological responsses, and have negative effects on health [3]. Older individuals are particularly prone for loneliness, because aging creates situations such as widowhood, mobility limitation, social exclusion, and living alone, that all increase opportunities for this feeling [4, 5]. Loneliness has been accepted as an independent health risk factor in later life and associated with a broad range of comorbidities, including aspects of mental, cognitive, and physical health, and impaired physical and daytime functioning [2–5]. Having theoretical framework to provide a viewpoint on complex associations between loneliness and poor health of older individuals, would help inform health promotion and disease prevention activities [3].

So far, a number of factors have been identified as associated with loneliness in older individuals. They can be grouped into several categories, including: 1. demographic (like advancing in age, female gender, poor education, low income, living alone, and being divorced or widowed) 2. environmental (like barriers to accessible housing or outdoor activities, low quality of relationships with others, and low social support), 4. physical or health-related (like increased number of chronic diseases, low mobility and other geriatric conditions, depression and other mental disordes), and 5. psychological factors (like low satisfaction with life, impaired well-being, poor emotion regulation control, and low perceived self-efficacy), in addition to 5. decreased functional abilities (daily functioning) [1, 6, 7]. To limit the scope of these factors, loneliness is usually viewed from two major perspectives − as social and emotional [8]. Social loneliness refers to an individual`s lack of engagement in broader social groups and proposes environmental factors. Emotional loneliness refers to the absence of close persons, a partner, relatives or friends, or to perceived lack of close emotional attachment, and involves psychological factors. A distinction between the two allows better understanding of the complex structure of the concept of loneliness and helps elaborate evaluation instruments.

A recent systematic review of longitudinal studies of risk factors for loneliness in older individuals has revealed a total of 120 risk factors examined, but only a few hold stability across the studies [9]. Those were mainly factors from the environmental domain, in addition to poor self-perceived health and depressed mood. In contrast to higher stability of demographic and environmental factors across studies and settings, their association with loneliness was weaker than that of physical (health-related) factors, while factors indicating psychological distress, in addition to widowhood, were shown as the most important predictors of loneliness [6].

**Fig. 1:**
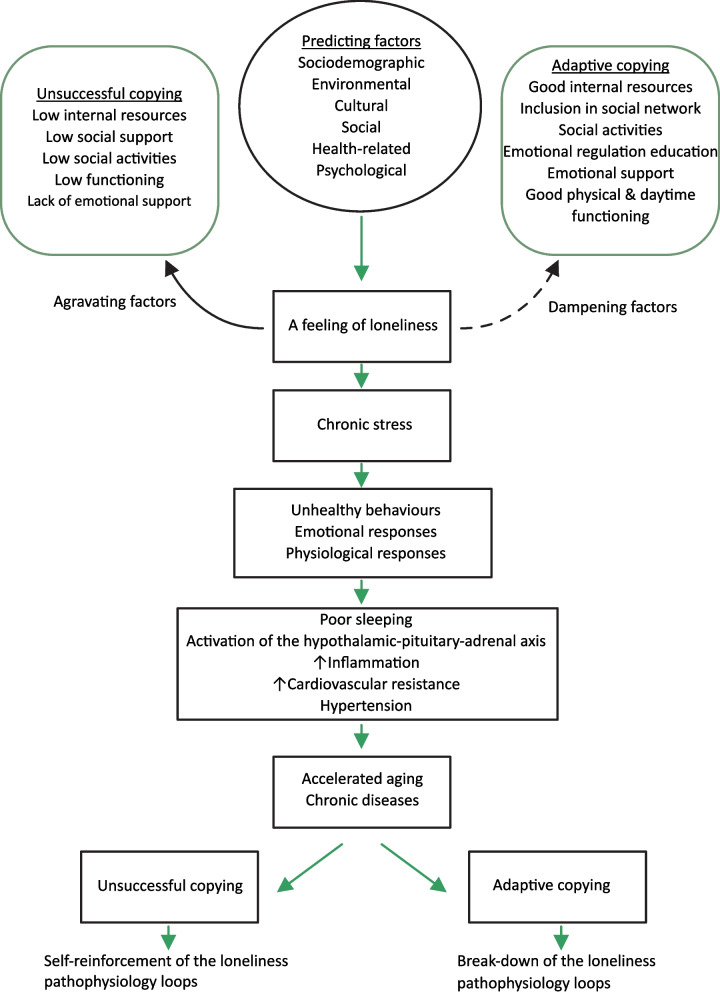
Model of loneliness in older individuals

Loneliness is increasingly viewed as a mediator in associations between different predisposing factors and poor health of older individuals [10]. The pathways proposed to link these associations include unhealthy behavioral and psychological responses, low sleep duration and quality, disturbed activity of the hypothalamus–pituitary–adrenal stress axis, an increase in cardiovascular resistance and blood pressure, changes in immune reactions, and increased inflammation (Fig. 1) [2, 11, 12]. Loneliness is supposed to initiate or maintain chronic stress mechanisms, which in individuals with low psychosocial resources (low psychological resilience and/or social support) and/or with inadequate coping with stress strategies, can accelerate aging and development of chronic diseases [13, 14].

It is not only that loneliness accelerates health deterioration, but also poor health may potentiate the feeling of loneliness. Having chronic diseases diminish one`s functional capabilities and opportunities to achieve desired levels of social activities, which can potentiate the feeling of loneliness [14, 15]. Adaptation to chronic diseases is particularly difficult in older age, when chronic diseases tend to accumulate, so that two or more chronic diseases usually coexist in one person, which is termed comorbidity [15]. An unsuccessful coping with”living with chronic diseases” erodes the one`s perception of self-sufficiency and diminishes self-esteem, and may cause emotional distress, which in turn may change the one`s perception of the stressful situation and/or of the availability of social support. This, finally, diminishes the one`s internal psychological resources (resilience), maintaining and/or aggravating the feeling of loneliness (Fig. 1) [14, 16].

**Fig. 2:**
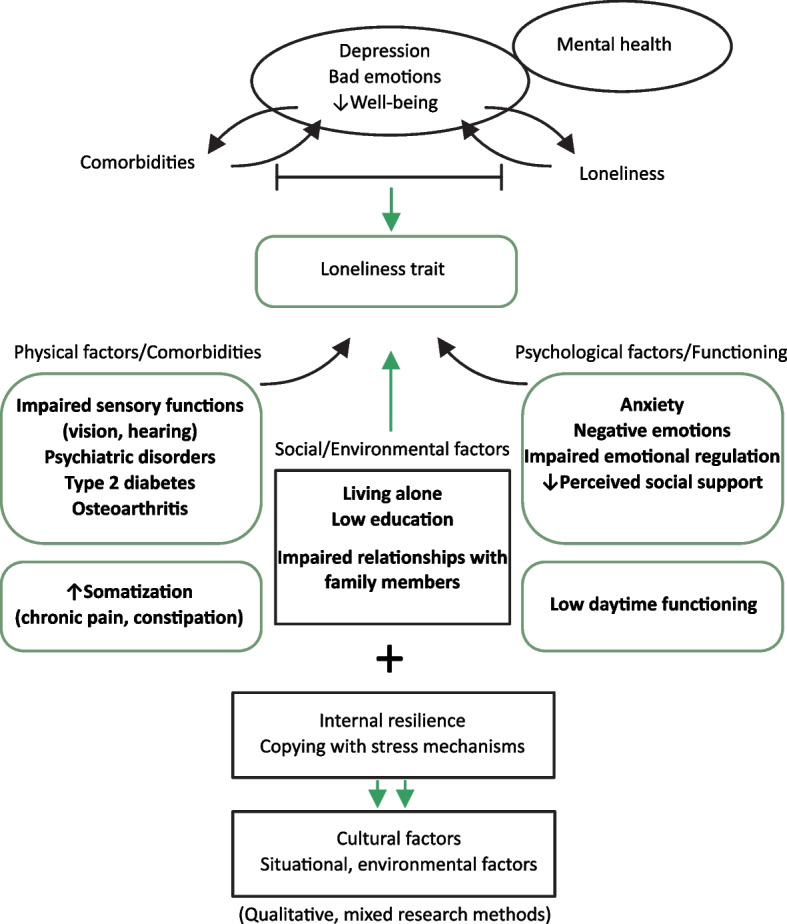
Loneliness trait – theoretical framework

Such course of aging can lead to the development of the “loneliness trait” – a profile of older individuals who suffer from loneliness, outlined by the set of specific psychosocial characteristics and comorbidity patterns, and relatively stable over time (Fig. 2) [3, 16]. To know this profile, it would help organize health-related preventive interventions. A prerequisite is an integrated model of loneliness in older individuals that could provide information on the role of particular factors that are involved in creating this trait, their interactions, and relative contributions [10]. Information is particularly scarce on the role of psychological factors in mediating associations between loneliness and poor health, despite the fact that these factors have been recognized as to have the highest impact on sustaining threats of being alone, and thus on reinforcing the loneliness-related pathophysiology loops [2].

Knowing characteristics which individuals suffering from loneliness have, and how to improve their psychosocial resources and/or to alleviate detrimental effects on health of negative emotions that usually accompany loneliness, such as hostility, distress, pessimism, anxiety, and low self-esteem, can slow down development of comorbidities, while enabling promotion of active aging [3, 17].

The aim of this study was to fill in some of these knowledge gaps, by attempting to create an integrated model of loneliness in older individuals burdened with comorbidities, considering different type of factors and their relative contributions to the trait loneliness, including: 1. demographic, 2. environmental, 3. physical (health-related) and 4. psychological, in addition to 5. functional abilities. In particular, we wanted to identify chronic health conditions with the highest impact on loneliness, and to assess the role of social and emotional perceptions and appraisals, as well as functional abilities, in mediating the link between poor health (the presence of comorbidities) and loneliness. We proposed that such integrated research approach can provide a theoretical framework for intervention optimization, which could alleviate the feeling of loneliness in older individuals, despite the presence of comorbidities and poor health.

## Methods

### Study design and participants

The data collection procedure was conducted during a period of 1.5 year, in a primary care (PC) setting, in the town of Osijek (approximately 60. 000 citizens) – a capital of the largest county in eastern Croatia. It lasted from August 2018 to January 2020, with variable intensity, being abruptly cut off by the outbreak of the COVID-19 pandemic in Europe, which in Croatia was announced in February 2020. Thus, the COVID-19 pandemic did not affect the data. The project was funded by the University of Osijek and approved by the Ethics Committee of the Faculty of Medicine, of the Josip Juraj Strossmayer University of Osijek (No. 641–01/18–01/01).

Participants (*N* = 189, F = 110) (mean age = 78.47 years, SD = 6.65) were recruited by two general practice (GP) teams, members of the teaching basis of the Faculty of Medicine Osijek, upon their agreement. These GP teams work in the urban area of the town of Osijek and provide care for about 4.000 patients in a total. PC patients in Croatia reflect well the structure of the adult general population of the local area, since citizens in Croatia have a free access to PC, and almost all have regulated compulsory health insurance status. The GP team leaders were provided with detailed information about the planned project and the data collection methods.

Participants were recruited by consecutive sampling, during their visits for other reasons. Included were individuals older than 60 years and who were able to independently come to their doctors and fill out the questionnaires. That means that they were independent of care of others and without visible cognitive impairments or severe physical disabilities (inability to walk independently, blind persons, persons with postictal aphasia, with uncorrected severe hearing loss, device dependent). Excluded were also patients with acute illnesses, with severe mental illnesses to whom communication is difficult, as well as those with some severe chronic conditions, like terminal or palliative patients and those on the permanent dialysis programme. Informed consent was obtained from all eligible individuals who agreed to participate. Before signing the informed consent form, they were informed of the purpose of the questionnaires.

All requested items and questionnaires were placed on the google platform, as the uniform survey, that was accessible through the link https://goo.gl/forms/uZlZ1NefjpQEGq9j2. A respondent was placed in the silent room, the door next to the GP, and had time as much as he/she needed for the survey. An educated administrator monitored the procedure and helped respondents fill out the questionnaires. The GP was easily available for any query.

### Data

A large set of data was collected to provide a comprehensive review of factors known to associate with loneliness in older individuals. The data was divided into categories, like: 1. demographic, 2. environmental, 3. physical (health-related) and 4. psychological factors, in addition to 5. functional abilities (Tables 1, 2, 3, 4 and 5). The demographic data and the data indicating physical (health-related) factors was used from patient electronic health records and checked during an interview with the patient. Information on environmental factors was used by an interview. Psychological characteristics and functional abilities were assessed by testing patients with the set of standard tests.

**Table 1:** Participant demographic characteristics

Characteristics	Levels	Number	%
Education	No education	1	0.53%
	Primary	43	22.75%
	Secondary	106	56.08%
	Tertiary	39	20.63%
Do you live alone	No	139	74%
	Yes	50	26%
Who do you live with?	Child	7	5%
	Children and their family	31	21%
	Foster family	2	1%
	Partner	9	6%
	Spouse	98	67%
Sexual activity	Active	34	18%
	Inactive	155	82%
Depression in early age	No	158	84%
	Yes	31	16%
Close family member mental disorders	No	167	88%
	Yes	22	12%

**Table 2:** Environmental factors (relationships with the close ones and neighbor, alcohol addiction)

Characteristics	Levels	Number	%
Difficulties in relationships with close family members	No	157	83%
	Yes	32	17%
Neighbour relationships	Very good	87	46%
	Good	87	46%
	Weak	12	6%
	No relationships	3	2%
How often do you consume alcohol	Often	5	3%
	Sometimes	50	26%
	Rarely or never	134	71%

**Table 3:** Environmrntal factors (hobbies/activities/social organization membership)

Hobby/activity	Number of participants engaged	%
Reading	81	42.86
Poem writing	3	1.59
Dance/folklore	7	3.70
Drawing	5	2.65
Crafts	48	25.40
Professional baking	1	0.53
Fishing	10	5.29
Gardening	5	2.65
Beekeeping	4	2.12
Sports	16	8.47
Social or religious organizations	15	7.94
No hobbies	49	25.93

**Table 4:** Physical factors (diagnoses of chronic diseases and geriatric conditions)

Diagnoses/geriatric conditions	Number of participants	%
Type 2 diabetes	37	19.58
Hypertension	160	84.66
Cardiovascular disease	89	47.09
Cerebrovascular disease	32	16.93
Severe osteoarthritis	118	62.43
Osteoporosis	28	14.81
Incontinentio urinae and other urinary bladder disorders	31	16.40
Chronic obstructive pulmonary disease	13	6.88
Chronic pain complaints	95	50.26
Upper gastrointestinal tract disorders	46	24.34
Constipation	46	24.34
Significant visual loss	41	21.69
Registered hearing impairment or communication difficulties due to hearing loss	43	22.75
Psychiatric diagnosis	45	23.81

**Table 5:** Standard scales used in the study (indicating loneliness, psychological factors and functional abilities)

	Mean	SD	Minimum	Maximum	Skewness	Kurtosis	α
UCLA	42.90	11.23	28	65	0.12	-1.44	.876
MSPSS_so	22.32	5.09	4	28	-1.51	1.85	.965
MSPSS_fam	22.17	5.11	4	28	-1.48	1.82	.967
MSPSS_fri	21.68	5.14	4	28	-1.13	0.84	.970
MSPSS_total	66.17	14.72	12	84	-1.40	1.83	.982
GAS	15.85	5.09	10	32	0.75	0.09	.925
GDS_dys	1.23	1.91	0	6	1.38	0.54	.883
GDS_pos	0.94	1.48	0	4	1.20	-0.22	.896
CA	2.94	4.65	0	28	2.53	8.68	.812
ADL	5.33	1.47	0	6	-2.55	5.79	.876
IADL	6.89	1.74	0	8	-1.78	3.00	.825
GSE	29.35	7.13	9	36	-0.66	-0.68	.990
ERQ_cognitive	28.89	6.42	13	42	0.02	-0.39	.964
ERQ_expressive	19.14	4.19	8	28	0.14	-0.44	.936

*UCLA* UCLA loneliness scale, *MSPSS_so* Multidimensional Scale of Perceived Social support- Signifficant other, *MSPSS_fam* Multidimensional Scale of Perceived Social support-Family, *MSPSS_fri* Multidimensional Scale of Perceived Social support- Friends, *MSPSS_total* Multidimensional Scale of Perceived Social support- Total score, *GAS* Geriatric Anxiety scale, *GDS_dys* Geriatrc Depression Scale- dysphoria, *GDS_pos* Geriatric Depression Scale- lack of positive mood, *CA* the 6-item cognitive impairment test indicating cognitive ability, *ADL* Katz Index of Independence of Daily Living, *IADL* Lawton-Brody Instrumental Activities of Daily Living Scale, *GSE* General Self-Efficacy Scale, *ERQ_cognitive* Emotional Regulation Quiestionnaire- Cognitive Reappraisal; Emotional Regulation Questionnaire- Expressive Suppresion

To get insights into different aspects of loneliness in the examined patients, we used a battery of standardized questionnaires, as a combination of instruments for testing negative emotions, psychological reactions, and functional abilities.

To screen patients on loneliness, we used the UCLA Loneliness Scale [18]. To assess patients on the presence of negative emotions, we used the Geriatric Anxiety scale (GAS) and the Geriatric Depression Scale (GDS) [19, 20]. In this regard, depression and anxiety are known as the common mental health disorders among older population; associations of loneliness with anxiety and depressive symptoms are stable in most cross-sectional and longitudinal studies [21]. To screen patients on cognitive function impairment, we used the 6-item cognitive impairment test, in this study termed as cognitive ability (CA) test [22]. We chose this test following evidence that loneliness, and not living alone or social isolation, has an impact on cognitive function decline [4, 23]. Actually, there is a bidirectional association between loneliness and impaired cognitive functions with the mediating role of impaired control of executive functions [24, 25]. The Katz Index of Independence of Daily Living (ADL) and Lawton-Brody Instrumental Activities of Daily Living Scale (IADL) were used to test the level of functional decline [26]. Functional decline is described as the loss of an individual’s ability to independently and safely perform activities of daily living, such as bathing, dressing, and eating, which is the basic level of daily activities, and activities such as shopping, driving, and banking, that indicate a higher level of performance [27]. Loneliness is associated with more rapid progression in both functional and motor decline, than expected for age [28, 29]. Although evidence on mechanisms linking loneliness with functional and motor decline is limited, it is assumed that psychosocial factors, such as poor self-regulation (executive control), impaired cognition, and increased somatization (associated with higher level of anxiety and chronic pain syndrome), have a significant role [29–31]. In addition, the feeling of loneliness changes the perceived availability of other persons for support, which may increase the level of stress and decrease the actual level of physical performance [32, 33]. We used the Multidimensional Scale of Perceived Social Support (MSPSS), as a measure of impaired ability to cope with challenges, in the context of lonelines [34]. In the panel test, we included also the Emotional Regulation Questionnaire (ERQ) [35]. We have been guided by the evidence that difficulties in emotion regulation is an important mechanism that can explain the observed association of loneliness with low adaptation abilities to environmental challenges, and behavioural and emotional problems, such as unhealthy lifestyles, anxiety, and depression [36]. The purpose that guided us to use of the General Self-Efficacy Scale (GSE) was that this test is considered as a measure of an intention for behavioural change, and is negatively correlated with negative emotions [37, 38].

### Descriptions of the instruments

The UCLA Loneliness Scale, version 3, that we used to measure the feeling of loneliness, is simply for use and thus appropriate for the elderly population [18]. Since this test has not been used before in the Croatian population, English version was translated into Croatian by three independent GPs, who then achieved a consensus on the translated version. The GP living in *Croatia,* whose mother tongue was English, translated it back to English (forward and backward translations). This scale contains 20 items describing different emotions associated with loneliness, with ten items worded in a negative direction, and 10 items worded in a positive direction. The items are scored on a 4-point scale, as: never, rarely, sometimes, and often. The coefficient of test reliability varies between 0.89 and 0.94 and the test-re-test reliability over a one-year period was *r* = 0.73.

The GAS and GDS tests, used for screening participants on anxiety and depression, are suitable for use among older population, as based on the ability to discriminate well between symptoms of mental, cognitive, and physical disorders [19, 20]. For these tests, we performed forward and backward translations, and cultural and linguistic validations. That is, the Croatian versions were sent to six GPs from different areas of Croatia, with request to indicate any lack of clarity in the translated statements. We also asked ten patients to check the tests and declare any misunderstanding. By taking all comments together, the research team then developed the final Croatian versions. For these tests, we estimated the internal factor structures, using the confirmatory factor analysis and several fit-of-model indices. We verified the stability of the identified domains by repeating the same procedure on similar patient sample from the parallel study [15, 39]. The best-fitted model for the GAS test was mono-dimensional 10-item model, whereas for the GDS test, it was two-dimensional 10-item model, with two factors termed as “dysphoria” and “the absence of positive mood” (GDS-pos.). The both tests provided a good fit of data, as indicated by the Cronbach`s alpha coefficients of 0.82 for the GAS test, and 0.81 and 0.80 for two domains of the GDS test.

The MSPSP consists of 12 items, and the translation and adaptation into Croatian has been performed before [40]. The result can be expressed as a total score of the sum of the responses on all particles, ranging from 12 to 84. A score in the range of 12 to 48 is considered a low level of perceived social support, a score in the range of 49 to 68 is considered a moderate level, and a score in the range of 69 to 84 is considered a high level of perceived social support. The results can also be analyzed through three subscales with four particles, these subscales called as: a family, friends, and significant others. A higher score on a particular subscale indicates a higher level of perceived social support. The internal consistency coefficient for the whole scale in previous studies was shown to be 0.93, while the reliability of the three subscales was ranging from 0.89 to 0.91 [41].

The 6-item cognitive impairment test (CA) is brief and simple, and practical for use in PC, for screening older individuals on mild dementia [42]. Compared with the standard and broadly used Mini-Mental State Examination (MMSE) test, this test is culturally unbiased and more sensitive for detecting people with mild cognitive impairments. The problem for the routine use of this test is limited availability of validation studies [22].

The ERQ measures respondents’ tendency to regulate emotions in two ways: cognitive reappraisal, and expressive suppression [35]. The capacity to control emotions is important for human adaptation. Respondents have to answer each of 10 items on a 7-point Likert-type scale ranging from 1 (strongly disagree) to 7 (strongly agree). Croatian adaptation and conceptual validation was performed by Gračanina and Kardum (2020) [43].

Believes in self-efficacy is a necessary pre-requisite for lifestyle changes, and is therefore an important target in health improvement programs. The GSE is a 10-item psychometric scale, designed to assess self-beliefs to cope with a variety of difficult demands in life [37]. The GSE is positively correlated with optimism, positive emotions, and work satisfaction, and negatively with depression, stress, health complaints, burnout, and anxiety. The response options are presented along a 4-point Likert-type scale for each item. The total score is calculated by finding the sum of all items and ranging between 10 and 40, with higher score indicating higher self-efficacy. In samples from 23 nations, Cronbach’s alphas ranged from 0.76 to 0.90.

Two related scales, ADL and IADL, measure physical self-maintenance, by items describing the one`s level of functioning in daily living [26]. Both tests were translated to Croatian by using forward and backward translations, and we performed cultural and lingvistic validation for these tests, too. The item “Ability to Use Telephone “, in IADL, was modified by the item “Ability to use telephone or mobile phone “. For ADLs, the total score ranges from 0 to 6, and for IADLs, it ranges from 0 to 8. In some categories, only the highest level of function receives a score of 1; in other categories, two or more levels have a score of 1, because each describes a competence that represents some minimal level of function.

### Analytical approach

All analyses were conducted in R v.4.0.3. [44]. Categorical variables were presented as absolute and relative frequencies (Tables 1, 2, 3 and 4). Descriptions of scales used in the study were provided as mean and SD (Table 5). Skewness (SI) and kurtosis (KI) indices (according to Kline) were used to indicate deviation from normal distribution; recommended values should not exceed 3 and 8, respectively [45]. Frequencies of UCLA scale scoring were provided in Table 6.

**Table 6:** Frequencies of UCLA scale scoring

UCLA scaling	Counts	% of Total	Cumulative %
Low	59	31%	31%
Moderate	51	27%	58%
Moderately high	77	41%	99%
High	2	1%	100%

Differences in particular variables, indicating demographic, environmental, and physical (health-related) factors (diagnoses of chronic health conditions), by the level of loneliness, were tested with Welch’s t-test and one-way ANOVA (Supplementary files 1, 2, 3 and 4). Welch’s t-test was chosen since there were large differences in compared group sizes. Differences were interpreted in terms of statistical significance at *p* < 0.05, and effect size values were estimated with Cohen’s d index [46]. Cohen’s d is an effect size measure, which standardizes mean difference between groups. Typically, d = 0.20 is interpreted as a small effect size, d = 0.50 is interpreted as a medium effect size, and d = 0.80 or larger is interpreted as a large effect size. The post-hoc Games-Howell test was used to differentiate between the levels of education.

In the next step, we inspected multicollinearity between variables used as potential predictors, and following recommendations, we excluded those with VIF values higher than 2.5 [47]. Because of indicated multicollinearity (VIF > 2.5), some scales were excluded from further analyses. Those scales were: GDS dysphoria (VIF = 2.86), ADL (VIF = 3.72), GSE (VIF = 2.62) and ERQ cognitive (VIF = 5.50). Intercorrelations were performed between loneliness (UCLA test), comorbidity level, and scales that were selected by multicolinearity analysis, using r- Pearson correlation coefficient (Supplementary file 5).

Next, we conducted hierarchical regression to explore relations of the groups of potential predictors with loneliness (Supplementary file 6) [48]. This procedure is a special form of multivariable regression, in which predictors are added in “blocks”, to examine if and in what amount blocks of predictors improve prediction of the outcome variable, loneliness in this case. Hierarchical regression was conducted in three steps. In the first step, there were categorical variables indicating education (primary, secondary or tertiary), living conditions (alone or with someone) and having hobbies (no hobbies or some hobbies), that were selected based on the analysis of demographic and environmental factors as control variables. In the next block, we entered physical factors (expressed as the level of comorbidity or the number of chronic health conditions) as a sole predictor. In the last, third block, we entered previously selected standard scales: MSPSS, GAS, GDS-pos., CA, IADL and ERQ-exp. Fit of successive regression blocks were interpreted by using ANOVA, calculating difference in models’ R^2^, and interpreting change in models’ Bayesian Information Criteria (BIC) [49]. When fitting models, it is possible to increase the likelihood by adding new variables, but it may result in overfitting. The BIC resolves this problem by introducing a penalty term for the number of parameters in the model. The lower the value of this measure, the better the model.

As the last step, we analyzed if any of participant characteristics, assessed by using standard scales, mediate relationship between health status, expressed as the level of comorbidity, and loneliness (Supplementary file 7). A mediator explains a way in which independent variable affects dependent variable, and here we seek for those variables that further explain relationship between comorbidity and loneliness, to shape further intervention recommendations. Potential mediators were included independently, by controlling for other independent variables, like education, living manner and hobbies, and effects from mediation analyses were estimated using 10.000 bootstrap simulations.

## Results

### Demographic and environmental factors

A total of 189 participants (110 or 58% women) older than 60 years (mean age 78.47 years, SD 6.65) took part in this study. There was no difference in age between men and women (*p* = 0.45). Participant demographic characteristics are provided in Table 1.

It is seen in Table 1 that most of participants had a secondary school (106 or 56.08%), and equal parts had a primary school (43 or 22.75%) and higher education (39 or 20.63%). One participant had not gone to school. About a quarter (50 or 26%) of participants stated that they are living alone. Of those who have not been alone, most were living with their spouses (98 or 67%), then follow in order of frequency those who were living with children and their families (31 or 21%), with a partner (9 or 6%), with a child (7 or 5%), while 2 participants (1%) were placed in a foster family. A large proportion of participants (155 or 82%) stated that they are not sexually active. A part of participants had depression in early age (31 or 16%) and a part of them reffered mental disorders in their close family members (22 or 12%).

Of environmental factors, we assessed relationships with the close ones and neighbor, alcohol addiction (Table 2), engagement in hobbies or other activities of a leisure time and a membership in social and religious organizations (Table 3).

It is seen in Table 2 that the majority of participants did not have difficulties in relationships with their close ones (157 or 83%) and that they maintained good relationships with neighbor (174 or 85%). Only 5 participants (3%) stated that they consume alcohol frequently (Table 2).

It is seen in Table 3 that a part of participants had no hobbies or activities (49 or 26%). Most frequent activities they were engaged in, were reading and crafting (42.86% and 25.40%, respectively), while the least used ones were professional baking and poem writing.

As physical factors, we used information on 14 common aging chronic diseases or diseases and geriatric conditions that are associated with disabilities (Table 4). Most frequent diagnoses/geriatric conditions were hypertension and severe osteoarthritis, while the least frequent one was chronic obstructive pulmonary disease.

### Scales description (psychological factors and functional abilities)

Scales used in the study indicate loneliness (UCLA), psychological factors (MSPSS, GAS, GDS, CA, GSE, ERQ) and functional abilities (ADL, IADL). Indices of skewness and kurtosis of scales’ distributions did not point to major deviations from the normal distribution. Furthermore, all scales had high internal consistency coefficients (Cronbach's alpha > 0.8) (Table 5).

As seen in Table 6, about three quarters of participants had some levels of loneliness (moderate-high). Moderately high and high levels of loneliness were recorded in 42% of participants.

### Results of analysis of differences

We assessed differences by the level of loneliness, and provided the effect size values, for categorical variables indicating demographic, environmental, and physical factors (diagnoses of chronic diseases and geriatric conditions) (Supplementary files 1, 2, 3 and 4).

Of demographic factors, we used gender, living manner, sexual activity, depression in early days (Supplementary file 1) and education levels (Supplementary file 2). Of envitonmental factors, we used family difficulties and information on having hobbies/activities or participation in social or religious organizations (Supplementary file 3). We excluded variables that in the previous analysis were shown to have highly asymmetric distributions at the sample level, such as often alcohol use, weak relationships/the absence of relationships with neighbor, and mental disorders in close family members.

The effect size analysis showed that loneliness was higher among older individuals who were living alone, suffered from depression in early days (Supplementary file 1), and had only primary education (based on the post-hoc analysis) (Supplementary file 2).

The effect size analysis showed that participants who had difficulties in relationships with their family members experienced higher level of loneliness than participants without such difficulties. On the contrary, those who were engaged in hobbies or other activities were less lonely, than those who were not (Supplementary file 3). However, while some activities were shown to be helpful in lessening the feeling of loneliness, like crafting, or sports, some others, like reading and being engaged in social or religious organizations, were not shown useful.

Participants diagnosed with type 2 diabetes, cerebrovascular disease, severe osteoarthritis, incontinentio urinae and other urinary bladder dysfunctions, chronic pain, constipation, significant visual loss, hearing impairment, and psychiatric disorders, all reported higher levels of loneliness compared to those without these diagnoses (Supplementary file 4).

### Intercorrelations between loneliness, comorbidity level, and selected scales

As indicated by intercorrelations between scales used in the study, correlations between MSPSS subscales and MSPSS total score were almost 1, which means that subscales are redundant. Therefore, further correlational analyses were conducted without MSPSS subscales. Intercorrelations between UCLA test (indicating loneliness), physical (health-related) factors (comorbidity level), and scales that were selected by multicollinearity analysis, showed that all correlations were significant at *p* < 0.05, except between MSPSS and CA test. The comorbidity level was significantly correlated with loneliness (Supplementary file 5).

### Hierarchical multivariable regression for predicting loneliness

Results of the hierarchical multivariable regression model for predicting loneliness are presented in Supplementary file 6.

The first model was controlled for variables indicating demographic and environmental factors. Before conducting regression analyses, we excluded a participant who reported not having any school degree. In this model, variables that were selected as significant predictors were: education, living alone (yes or no), and having hobbies (yes or no). This model was significant (*p* < 0.001), explaining 15% of variance of loneliness.

In the second step, comorbidity level was introduced as a predictor, which led to the improvement in the model predictive performance for 16% (ΔR^2^ = 0.07, F (1, 182) = 16.11, *p* < 0.001). This variable was shown as a moderate positive predictor of loneliness.

In the third step, scales were introduced, including MSPSS, GAS, GDS-pos, CA, IADL, and ERQ-exp. This led to further improvement of the model likelihood for 27.53% (ΔR^2^ = 0.37, F (6, 176) = 27.53, *p* < 0.001). When adjusted with other variables, IADL lost significant correlation with loneliness. MSPPS, CA test, and ERQ-exp., were negatively correlated with loneliness, while GAS and GDS-pos. were positive predictors of loneliness. Among those predictors, anxiety (GAS test) was shown as the strongest predictor of loneliness.

The full model explained 58% of variance of loneliness. The comparison of models using BIC gives rise to the conclusion that by adding standard psychological tests to the pre-existed models, representing demographic, environmental, and physical factors (comorbidity level), this improves model`s goodness of fit and also the model`s accuracy.

### Mediation analysis

Mediation analysis informs us how we can help patients with comorbidities to feel less lonely. Results of exploring participant characteristics assessed by using standard scales as potential mediators in relationships between comorbidity level and loneliness are presented in Supplementary file 7.

As seen in Supplementary file 7, CA was the only tested mediator that did not show significant mediation effect. On the other hand, MSPSS (β = 0.08), GAS (β = 0.18), GDS-pos. (β = 0.14), IADL (β = 0.09), and ERQ-exp. (β = 0.13) were shown as significant mediators. The results suggest that it is possible to alleviate loneliness by including interventions that would reduce anxiety (GAS) and depression (GDS-pos.) and improve emotional regulation (ERQ-exp.) or perceived social support (MSPSS).

## Discussion

This study explored relation between impaired health status and loneliness in older individuals in an integrative way. Results indicated that increasing level of comorbidity, anxiety, lack of positive moods, not having hobbies/activities, low subjective perception of social support, impaired cognitive function, and suppression of emotion expression, are all significant predictors of loneliness. Negative emotions and poor emotion regulation control were highlighted as the main mediating mechanisms in this relation. The results are important from the translational perspective.

Although participants were older individuals with multiple chronic health conditions (mean 4.36, SD 2.58), they were able to visit their GPs independently, and their functional abilities and cognitive functions were shown pretty good (IADL and CA tests). In line with these characteristics, and the fact that they maintained good neighbour and within family relationships, their perceived social support was also good (high score on MSPPS test). Less consistent with these results, and by taking into account also the fact that about a quarter of participants were living alone, was the finding on high rates of loneliness (about 70%), with more than 40% of participants showing higher levels of loneliness. This is much more than what was reported for the general population in EU countries, where the maximal rate before COVID-19 pandemic mounted 20%, and also for Croatia (about 10%). Even during the COVID-19 pandemic, the maximal rates in EU countries did not exceed 26% [50].

For reasonable explanations, we should search in the sphere of emotions and emotion regulation control, as pillars of the definition of loneliness, which in the context of aging and multiple comorbidities might be greatly disturbed. Such an impression arises from the result indicating negative emotions and inadequate emotion regulation as the main mechanisms mediating the link between comorbidity level and loneliness, and the fact that negative emotions and an increasing level of comorbidity were shown as the strongest positive predictors of loneliness (regression model).

To our knowledge, this is the first attempt to use an integrated approach to show how different types of factors work together to predict loneliness. Previous research revealed many factors as associated with loneliness, but they were taken solely, out of the context. This led to the semantic rather than the nosologic classification of these factors, and to the dichotomy between social and emotional loneliness. This approach, however, might be inssuficient today, when loneliness is confirmed as an independent risk factor for some pillars of aging, such as depression, cardio-metabolic conditions, and dementia, which argues for focused and efficient interventions [51–53]. In addition, neurobiological pathways have been identified in the brain that are associated with the disrupted affective processes that typically characterize individuals experiencing loneliness, thus highlighting the biological and psychological mechanisms as to be inextricably linked together [54].

This study is the first one to comply with these requirements. Overall, we found that disturbed health (an increasing level of comorbidity), and demographic and environmental factors, contribute eaqually to the variance of loneliness. Variables that contributed the most were those from the psychological domain, including impaired social and emotional perception and appraisals (MSPSS and ERQ-exp tests) and negative moods (GAS and GDS-pos. tests). This means, also, that the most effective interventions would be that from the psychological domains. In addition, only through interactions of different factors it would be possible to recognize pathways that stay in the background of loneliness and poor health, and how they change with variations in characteristics of the target populations.

The results of this study are presented in Fig. 2 in a condensed way. This model of loneliness trait in elderly persons reveals a highly complex structure, with negative emotions and impaired emotion regulation being in the centre of the loop that links loneliness and poor health (comorbidities). The available evidence supports this picture. In elderly population, physical and psychological disorders are known to be closely related, with inflammation being the common denominator [55–57]. In this regard, pro-inflammatory cytokines, that are produced in excess in the context of chronic aging diseases, can change the neurobiological pathways in the brain, which can disrupt regulation of affective processes, potentiating or aggravating the feeling of loneliness [58, 59].

The opposite is also true. Bad emotions can act as chronic stress mechanisms, which through an array of physiological mechanisms diminish homeostatic resources and leads to alostatic load states, thus ultimately accelerating health deterioration [60]. In addition, and especially in individuals with pre-existing low psychological resilience (such as those with mental health problems lasting from younger age or those who pretend somatic reactions to chronic stress) (see also Fig. 2), bad emotions can aggravate these pathological pathways. One way is by changing the one`s perception and appraisal of the stressful situation as being more frightening [17, 59]. This way, by turning the focus of an individual to emotional reactions, his/her ability to manage daily activities, and thus also to care on healthy lifestyle choices, may also attenuate, which can further negatively influence the health [17].

As visible in Fig. 2, and also suggested by the evidence, the scenario linking loneliness and poor health in older individuals is even more complex than presented above. Not only anxiety/depression, but also a broad array of negative emotional reactions that usually occupy older individuals burdened with chronic diseases and functional deficits, such as the loss of self-confidence and of the sense of purpose, demoralization, and the fear of becoming disabled, may distort their perceptions, and help maintain chronic stress mechanisms active [61]. When viewed from this perspective, the scope of diagnoses, selected in this study as to associate with loneliness, becomes easily understandable (Fig. 2). These disorders, sensory organ impairment, and some common age-related conditions, like type 2 diabetes, cerebrovascular diseases, and osteoarthritis, are all known as highly debilitating and/or associated with increased inflammation, and are all cited in the literature as associated with loneliness [62–64].

If not currently disabled, older individuals may experience discomfort associated with the fear of upcoming disabilities [65]. This fear might be a key point to explain a discrepancy between good functional abilities of participants in this study and their good perception of social support, and high prevalence of loneliness, on the other side. Important to mention is also the fact that bad emotions in older individuals may additionally be powered by neurobiological mechanisms of neuroinflammation and neurovascular disorders, that in the brain develop in association to aging and the presence of chronic diseases [59, 66–68].

In this regard, evidence suggets that mental disorders in older individuals usually appear in the context of existing comorbidities, rather than as sole (“real “) psychiatric diseases [55]. This is the reason that symptoms of mental disorders often overlap with those of cognitive dysfunctions and physical conditions, including a large array of nonspecific reactions, such as discomfort, tension, emotional blunting, worry, disturbed sleep, irritability, and fatigue [69, 70]. Results of this study also support close associations of physical and mental disorders in older individuals, as suggested by the result indicating that also comorbidity level, anxiety (GAS), depressive moods (GDS-pos.), and cognitive function (CA), are predictors of loneliness, and the fact that psychiatric disorders take part in the pattern of chronic diseases that are associated with loneliness.

One more explanation for the high level of expression of loneliness in this vulnerable population is also suggested by these results. This is a predisposition of these participants, burdened with comorbidities, for somatization – a mental disorder defined as transmission of mental discomfort into physical symptoms (Fig. 2) [71]. This is suggested by their only mildly impaired cognitive function and the fact that cognitive dysfunction was not indicated as a mediator in association between comorbidity and loneliness. Instead of that, functional disorders and psychosomatic symptoms, like chronic pain, constipation, and incontinentio urinae, were shown as important features of loneliness trait (Fig. 2) [72]. A hypothesis that arises, is that not just neurobiological pathways, but rather secondary upgrated mechanisms, such as somatization, may in older individuals with multiple comorbidities have a major role in pathways associated with emotion processing disregulation and the feeling of loneliness.

Associated with this hypothesis, and also supported by the results of this study, is another hypothesis. It states that the presence of chronic diseases and functional deficits in older individuals may be a more important source of chronic stress and a reason of emotional dysregulation associated with the feeling of loneliness, than living alone. It is suggested by a discrepancy between the number of participants who reported to live alone (a quarter) and a number of those who reported loneliness (three quartiers), and also by the results of the regression model, where variable “living alone “ has drastically lost its impact on loneliness (the outcome variable) when variables indicating health status (comorbidity level) and psychological disorders, were added to the initial model. Recent evidence from the lock-down period of the COVID-19 pandemic, supports this assumption, indicating that social isolation has higher effect on the prevalence of loneliness and mental health deterioration in younger adult groups, than in the elderly population [50, 73, 74].

Taken together, this study revealed an old true that psychological adaptation of older individuals to living with chronic diseases is challenging (Fig. 1) [75]. Specifically, the loop linking comorbidities, loneliness, and low emotion regulation, may be a major driving force on the course of “unhealthy aging “ (Fig. 2) [76]. Nevertheless, and as other authors also stated, factors associated with loneliness in older individuals are multi-faceted, which implies the need for an integrated research approach [77]. A fortunate thing is that new methods for data analysis, like network and mediation analyses, are widely available, and can be used to create complex models, to serve as theoretical frameworks for planning interventions. For more deeper insights into environmental and cultural factors of loneliness, and emotional experiences of older individuals suffering of loneliness, there will be also a need for qualitative and mixed research methods (Fig. 2) [78].

The key message of this study is yet that caring for mental health of older individuals should be in the centre of strategies aimed at both, alleviating loneliness, and protecting health and functional abilites of older individuals from accelerated deterioration (Fig. 2). For this purpose, some authors recommend rehabilitation programmes that will enhance competencies of older individuals for positive reframing [76, 79]. To the similar conclusion we came in our recently published paper, where we found that older individuals with good physical and mental functioning, despite the presence of chronic diseases, mostly use positive coping styles [80]. Results of this study fits into the same frame, respecting that having hobbies/activities of a leisure time was valued as an important protective mechanism of loneliness. For personalization of the therapy, it should take into account an individual`s pre-existing internal resources and personality characteristics (Fig. 2) [79, 81].

Of particular interventions, our results suggest activities such as crafting, that encourage motor skills, creativity, attention, and planning. Similarly, literature review provides evidence on the effectiveness of the mindfulness-based therapy for alleviating loneliness and improving everyday functioning of older individuals [82]. Because physical and mental resilience in older individuals are mutually related, some preventive measures from the physical health domain, such as healthy lifestyles, looks like to be useful also in promoting psychological resilience [17, 83]. Some activities, in contrast, mentioned in this study, such as reading or participating in social or religious organizations, may mearly be a sign of loneliness, rather than efficacious preventive measures, probably reflecting coping mechanisms such as negative appraisal of social company, or seeking for emotional relief and spiritual support [84]. Thus, the scope of measures that sholud be recommended to older individuals suffering of loneliness go far away beyond the narrow scope of interventions that are recommended today, which simply focus on increase in social interactions. Instead, interventions are also needed to target social and emotional perceptions and emotion regulation control, together with implementation of healthy lifestyle habits.

## Conclusions & limitations

This study represents an integrated model of loneliness in older individuals burdened with comorbidities, but who are still functioning well. Results are supposed to improve understanding of the pathophysiology loops linking comorbidities and loneliness and can be used to inform interventions. The study has several limitations. One is the bias in data collection, since only older individuals who came to their GPs for consultations were included in analysis. In addition, there could have been a bias in passing tests, as there was a large batery of tests, which could have been tiresome for older people. Also, there was a little control of variables in the methods. Future research should focus on optimization of the pool of variables that would be appropriate to enter the predictive model and on searching for comprehensive analytical methods that can be used to get replicative results.

### Supplementary Information

**Additional file 1: Table S1.** Differences in demographic variables (gender, living alone, sexual activity, and depression in early days) by status loneliness.

**Additional file 2: Table S2.** Differences in demographic variables (educational levels) by status loneliness.

**Additional file 3: Table S3.** Differences in environmental factors (family difficulties, hobbies/activities/social or religious organization participation) by status loneliness.

**Additional file 4: Table S4.** Differences in physical factors (diagnoses of chronic diseases and geriatric conditions) by status loneliness.

**Additional file 5: Table S5.** Intercorrelations between loneliness, physical factors (comorbidity level), psychological factors (psychological tests) and functional abilities (IADL).

**Additional file 6: Table S6.** Results of hierarchical multivariable regression for predicting loneliness.

**Additional file 7: Table S7.** Standard tests indicating participant psychological characteristics and functional abilities (IADL) as mediators in the relation between health status (comorbidity level) and loneliness.

## Data Availability

The datasets generated and/or analysed during the current study are not publicly available due to clinical data protection but are available upon request from Thomas Wittlinger and Majnarić Trtica Ljiljana.

## Abbreviations

GP: General practice
PC: Primary care
GAS: Geriatric Anxiety scale
GDS: Geriatric Depression Scale
CA: Cognitive ability test
ADL: Index of Independence of Daily Living
IADL: Instrumental Activities of Daily Living Scale
MSPSS: Multidimensional Scale of Perceived Social Support
ERQ: Emotional Regulation Questionnaire
