# Supplementary material for: Psycho-social and health predictors of loneliness in older primary care patients and mediating mechanisms linking comorbidities and loneliness
Source: BMC Geriatr. 2023 Dec 4;23:801. doi: 10.1186/s12877-023-04436-6 (PMC10696735; doi:10.1186/s12877-023-04436-6)
Supplement: Supplementary file 1 — Additional file 1: Table S1. Differences in demographic variables (gender, living alone, sexual activity, and depression in early days) by status loneliness. [file 12877_2023_4436_MOESM1_ESM.docx]

Table S1 Differences in demographic variables (gender, living alone, sexual activity, and depression in early days) by status loneliness

|  | Levels | N | M(SD) | t | df | p | Cohen's d |
| --- | --- | --- | --- | --- | --- | --- | --- |
| Gender | Female | 110 | 44.03 (11.22) | 1.63 | 169.01 | .105 | 0.24 |
|  | Male | 79 | 41.34 (11.13) |  |  |  |  |
| Living alone | No | 139 | 41.81 (11.20) | 2.30 | 89.24 | .024 | 0.38 |
|  | Yes | 50 | 45.96 (10.84) |  |  |  |  |
|  | Yes | 32 | 48.16 (10.59) |  |  |  |  |
| Sexual activity | No | 135 | 42.95 (11.79) | 0.11 | 46.83 | .913 | 0.02 |
|  | Yes | 34 | 42.71 (11.79) |  |  |  |  |
| Depression in early days | No | 158 | 41.78 (11.15) | 3.41 | 45.85 | .001 | 0.64 |
|  | Yes | 31 | 48.61 (10.01) |  |  |  |  |
