# Supplementary material for: Psycho-social and health predictors of loneliness in older primary care patients and mediating mechanisms linking comorbidities and loneliness
Source: BMC Geriatr. 2023 Dec 4;23:801. doi: 10.1186/s12877-023-04436-6 (PMC10696735; doi:10.1186/s12877-023-04436-6)
Supplement: Supplementary file 2 — Additional file 2: Table S2. Differences in demographic variables (educational levels) by status loneliness. [file 12877_2023_4436_MOESM2_ESM.docx]

Table S2 Differences in demographic variables (educational levels) by status loneliness

| Education | Levels | N | M(SD) | F | df | p | Games-Howell post hoc |
| --- | --- | --- | --- | --- | --- | --- | --- |
|  | Primary | 43 | 47.81 (9.60) | 2.11 | 2, 185 | .002 | Primary > Secondary (p = .004, d = 0.55), Tertiary (p = .004, d = 0.71) |
|  | Secondary | 106 | 41.83 (11.18) |  |  |  |  |
|  | Tertiary | 39 | 40.03 (11.49) |  |  |  |  |
