# Supplementary material for: Psycho-social and health predictors of loneliness in older primary care patients and mediating mechanisms linking comorbidities and loneliness
Source: BMC Geriatr. 2023 Dec 4;23:801. doi: 10.1186/s12877-023-04436-6 (PMC10696735; doi:10.1186/s12877-023-04436-6)
Supplement: Supplementary file 3 — Additional file 3: Table S3. Differences in environmental factors (family difficulties, hobbies/activities/social or religious organization participation) by status loneliness. [file 12877_2023_4436_MOESM3_ESM.docx]

Table S3 Differences in environmental factors (family difficulties, hobbies/activities/social or religious organization participation) by status loneliness

| Hobbies | Levels | N | M(SD) | t | df | p | Cohen's d |
| --- | --- | --- | --- | --- | --- | --- | --- |
| Family difficulties | No | 157 | 41.83 (11.09) | 3.05 | 45.93 | .004 | 0.58 |
| Hobbies/activities/organization participation |  |  |  |  |  |  |  |
| Do you have hobbies | No | 49 | 49.59 (8.72) | 5.79 | 106 | < .001 | 0.90 |
|  | Yes | 140 | 40 (11.09) |  |  |  |  |
| Reading | No | 59 | 37.22 (10.21) | 3.18 | 130.84 | .002 | 0.54 |
|  | Yes | 81 | 43.00 (11.14) |  |  |  |  |
| Crafts | No | 92 | 42.98 (11.43) | 4.04 | 118.64 | < .001 | 0.69 |
|  | Yes | 48 | 35.94 (8.81) |  |  |  |  |
| Fishing | No | 130 | 40.62 (11.27) | 0.24 | 11.37 | .814 | 0.07 |
|  | Yes | 10 | 39.90 (8.86) |  |  |  |  |
| Sports | No | 124 | 41.32 (10.90) | 2.25 | 19.91 | .037 | 0.60 |
|  | Yes | 16 | 34.69 (11.13) |  |  |  |  |
| Social or religious organizations | No | 125 | 39.82 (10.95) | 2.39 | 17.74 | .028 | 0.65 |
|  | Yes | 15 | 46.80 (10.65) |  |  |  |  |
