# Supplementary material for: Psycho-social and health predictors of loneliness in older primary care patients and mediating mechanisms linking comorbidities and loneliness
Source: BMC Geriatr. 2023 Dec 4;23:801. doi: 10.1186/s12877-023-04436-6 (PMC10696735; doi:10.1186/s12877-023-04436-6)
Supplement: Supplementary file 4 — Additional file 4: Table S4. Differences in physical factors (diagnoses of chronic diseases and geriatric conditions) by status loneliness. [file 12877_2023_4436_MOESM4_ESM.docx]

Table S4 Differences in physical factors (diagnoses of chronic diseases and geriatric conditions) by status loneliness

| Diagnosis | Levels | N | M(SD) | t | df | p | Cohen's d |
| --- | --- | --- | --- | --- | --- | --- | --- |
| Diabetes type 2 | No | 152 | 41.92 (11.18) | 2.55 | 56.87 | .014 | 0.46 |
|  | Yes | 37 | 46.95 (10.66) |  |  |  |  |
| Hypertension | No | 29 | 39.38 (10.35) | 1.97 | 41.06 | .056 | 0.38 |
|  | Yes | 160 | 43.54 (11.30) |  |  |  |  |
| Cardiovascular disease | No | 100 | 42.28 (11.55) | 0.81 | 186.36 | .416 | 0.12 |
|  | Yes | 89 | 43.61 (10.89) |  |  |  |  |
| Cerebrovascular disease | No | 157 | 42.16 (11.20) | 2.09 | 45.66 | .045 | 0.40 |
|  | Yes | 32 | 46.56 (10.80) |  |  |  |  |
| Severe osteoarthritis | No | 71 | 39.80 (10.90) | 3.02 | 149.26 | .003 | 0.45 |
|  | Yes | 118 | 44.77 (11.06) |  |  |  |  |
| Osteoporosis | No | 161 | 42.48 (11.25) | 1.25 | 37.46 | .218 | 0.25 |
|  | Yes | 28 | 45.32 (11.02) |  |  |  |  |
| Incontinentio urinae | No | 158 | 42.18 (11.38) | 2.23 | 47.44 | .031 | 0.41 |
|  | Yes | 31 | 46.58 (9.78) |  |  |  |  |
| Chronic obstructive pulmonary disease | No | 176 | 43.28 (11.23) | 1.86 | 14.21 | .084 | 0.51 |
|  | Yes | 13 | 37.77 (10.26) |  |  |  |  |
| Chronic pain | No | 94 | 39.91 (10.47) | 3.77 | 186.33 | < .001 | 0.55 |
|  | Yes | 95 | 45.86 (11.23) |  |  |  |  |
| Upper gastrointestinal tract disorders | No | 143 | 42.07 (10.88) | 1.72 | 70.33 | .089 | 0.30 |
|  | Yes | 46 | 45.50 (12.01) |  |  |  |  |
| Constipation | No | 143 | 41.38 (11.08) | 3.49 | 80.09 | < .001 | 0.58 |
|  | Yes | 46 | 47.65 (10.45) |  |  |  |  |
| Significant visual loss | No | 148 | 41.02 (10.74) | 4.69 | 65.43 | < .001 | 0.82 |
|  | Yes | 41 | 49.71 (10.42) |  |  |  |  |
| Hearing impairment | No | 146 | 41.63 (11.09) | 2.99 | 70.62 | .004 | 0.51 |
|  | Yes | 43 | 47.23 (10.72) |  |  |  |  |
| Psychiatric diagnoses | No | 144 | 40.52 (10.95) | 6.44 | 94.25 | < .001 | 1.02 |
|  | Yes | 45 | 50.53 (8.45) |  |  |  |  |
