# Supplementary material for: Psycho-social and health predictors of loneliness in older primary care patients and mediating mechanisms linking comorbidities and loneliness
Source: BMC Geriatr. 2023 Dec 4;23:801. doi: 10.1186/s12877-023-04436-6 (PMC10696735; doi:10.1186/s12877-023-04436-6)
Supplement: Supplementary file 5 — Additional file 5: Table S5. Intercorrelations between loneliness, physical factors (comorbidity level), psychological factors (psychological tests) and functional abilities (IADL). [file 12877_2023_4436_MOESM5_ESM.docx]

Table S5 Intercorrelations between loneliness, physical factors (comorbidity level), psychological factors (psychological tests) and functional abilities (IADL)

|  |  |  |  |  |  |  | r | | | | | | |
| --- | --- | --- | --- | --- | --- | --- | --- | --- | --- | --- | --- | --- | --- |
|  | M | SD | Min | Max | SI | KI | 1 | 2 | 3 | 4 | 5 | 6 | 7 |
| Comorbidity (1) | 4.36 | 2.58 | 0 | 12 | 0.65 | 0.00 |  |  |  |  |  |  |  |
| UCLA (2) | 42.90 | 11.23 | 28 | 65 | 0.12 | -1.44 | .40 |  |  |  |  |  |  |
| MSPSS (3)* | 66.17 | 14.72 | 12 | 84 | -1.40 | 1.83 | -.17 | -.55 |  |  |  |  |  |
| GAS (4) | 15.85 | 5.09 | 10 | 32 | 0.75 | 0.09 | .31 | .64 | -.41 |  |  |  |  |
| GDS_pos (5) | 0.94 | 1.48 | 0 | 4 | 1.20 | -0.22 | .26 | .58 | -.47 | .52 |  |  |  |
| CA (6) | 2.94 | 4.65 | 0 | 28 | 2.53 | 7.68 | .53 | .19 | -.13 | .20 | .31 |  |  |
| IADL (7) | 6.89 | 1.74 | 0 | 8 | -1.78 | 3.00 | -.46 | -.33 | .26 | -.27 | -.46 | -.60 |  |
| ERQ exp (8) | 19.14 | 4.19 | 8 | 28 | 0.14 | -0.44 | -.32 | -.50 | .36 | -.46 | -.38 | -.25 | .30 |

*Only scales were used selected by multicolinearity analysis.

GDS_pos – GDS - lack of positive emotions; ERQ exp - ERQ expression; M- Mean, SD- standard deviation;

Min- minimum value; max- maximum value; SI- skewness index; KI- kurtosis index;

r- Pearson correlation coefficient.
