# Supplementary material for: Psycho-social and health predictors of loneliness in older primary care patients and mediating mechanisms linking comorbidities and loneliness
Source: BMC Geriatr. 2023 Dec 4;23:801. doi: 10.1186/s12877-023-04436-6 (PMC10696735; doi:10.1186/s12877-023-04436-6)
Supplement: Supplementary file 6 — Additional file 6: Table S6. Results of hierarchical multivariable regression for predicting loneliness. [file 12877_2023_4436_MOESM6_ESM.docx]

Table S6 Results of hierarchical multivariable regression for predicting loneliness

|  | Block 1 | | | | | Block 2 | | | | | Block 3 | | | | |
| --- | --- | --- | --- | --- | --- | --- | --- | --- | --- | --- | --- | --- | --- | --- | --- |
|  |  |  | 95% CI β | |  |  |  | 95% CI β | |  |  |  | 95% CI β | |  |
| Predictor | B | β | Lower | Upper | p | B | β | Lower | Upper | p | B | β | Lower | Upper | p |
| Intercept | 43.39 |  |  |  | < .001 | 37.05 |  |  |  | < .001 | 46.05 |  |  |  | < .001 |
| Education | |  |  |  |  |  |  |  |  |  |  |  |  |  |  |
| Secondary | **-4.54** | **-0.40** | **-0.74** | **-0.07** | **0.017** | -2.60 | -0.23 | -0.56 | 0.10 | 0.168 | -0.79 | -0.07 | -0.32 | 0.18 | 0.579 |
| Tertiary | -4.59 | -0.41 | -0.83 | 0.01 | 0.054 | -2.18 | -0.19 | -0.61 | 0.22 | 0.356 | 0.58 | 0.05 | -0.27 | 0.38 | 0.751 |
| Living alone | **3.43** | **0.31** | **0.00** | **0.61** | **0.047** | 2.23 | 0.20 | -0.10 | 0.49 | 0.187 | 0.74 | 0.07 | -0.16 | 0.29 | 0.562 |
| No hobbies | **7.96** | **0.71** | **0.40** | **1.02** | **< .001** | **-6.68** | **-0.60** | **-0.90** | **-0.29** | **< .001** | -2.08 | -0.19 | -0.44 | 0.07 | 0.153 |
| Comorbidity |  |  |  |  |  | **1.24** | **0.29** | **0.14** | **0.43** | **< .001** | **0.94** | **0.22** | **0.10** | **0.34** | **< .001** |
| MSPSS |  |  |  |  |  |  |  |  |  |  | **-0.17** | **-0.22** | **-0.33** | **-0.11** | **< .001** |
| GAS |  |  |  |  |  |  |  |  |  |  | **0.66** | **0.30** | **0.18** | **0.42** | **< .001** |
| GDS-pos |  |  |  |  |  |  |  |  |  |  | **1.78** | **0.24** | **0.11** | **0.36** | **< .001** |
| Cognitive ability |  |  |  |  |  |  |  |  |  |  | **-0.41** | **-0.17** | **-0.30** | **-0.04** | **0.012** |
| IADL |  |  |  |  |  |  |  |  |  |  | -0.11 | -0.02 | -0.15 | 0.11 | 0.799 |
| ERQ-expressive |  |  |  |  |  |  |  |  |  |  | **-0.35** | **-0.13** | **-0.25** | **-0.01** | **0.027** |
| Model fit | |  |  |  |  |  |  |  |  |  |  |  |  |  |  |
| ANOVA | F(5, 183) = 8.03, p < .001 | | | | | F(6, 182) = 9.93, p < .001 | | | | | F(12, 176) = 23.07, p < .001 | | | | |
| R^2^ | .17 | | | | | .24 | | | | | .61 | | | | |
| Adj. R^2^ | .15 | | | | | .22 | | | | | .58 | | | | |
| BIC | 1437.11 | | | | | 1426.40 | | | | | 1333.39 | | | | |
| Model comparisons | |  |  |  |  |  |  |  |  |  |  |  |  |  |  |
| ΔR^2^ |  |  |  | .07 | | | |  | .37 | | | |  |  |  |
| ANOVA |  |  |  | F(1, 182) = 16.11, p < .001 | | | |  | F(6, 176) = 27.53, p < .001 | | | |  |  |  |

Note: referent categories: Education- “Primary”, Living alone- “No”; No hobbies – “Having hobbies”; MSPSS-total - Multidimensional Scale of Perceived Social support -Total score; GAS - Geriatric Anxiety scale; GDS-pos - Geriatric Depression Scale - lack of positive mood; IADL - Lawton-Brody Instrumental Activities of Daily Living Scale; Emotional Regulation Questionnaire - Expressive Suppression
