# Supplementary material for: Psycho-social and health predictors of loneliness in older primary care patients and mediating mechanisms linking comorbidities and loneliness
Source: BMC Geriatr. 2023 Dec 4;23:801. doi: 10.1186/s12877-023-04436-6 (PMC10696735; doi:10.1186/s12877-023-04436-6)
Supplement: Supplementary file 7 — Additional file 7: Table S7. Standard tests indicating participant psychological characteristics and functional abilities (IADL) as mediators in the relation between health status (comorbidity level) and loneliness. [file 12877_2023_4436_MOESM7_ESM.docx]

Table S7 Standard tests indicating participant psychological characteristics and functional abilities (IADL) as mediators in the relation between health status (comorbidity level) and loneliness

|  |  |  | 95% CI B | |  |  |
| --- | --- | --- | --- | --- | --- | --- |
| Mediator | Effect | B | Lower | Upper | β | p |
| MSPSS | Indirect | 0.35 | 0.03 | 0.66 | 0.08 | **0.031** |
|  | Comorbidity > MSPSS | -0.92 | -1.73 | -0.11 | -0.16 | 0.026 |
|  | MSPSS > UCLA | -0.38 | -0.46 | -0.29 | -0.49 | < .001 |
|  | Direct | 1.38 | 0.89 | 1.87 | 0.32 | < .001 |
|  | Total | 1.72 | 1.15 | 2.30 | 0.39 | < .001 |
| GAS | Indirect | 0.77 | 0.40 | 1.14 | 0.18 | **< .001** |
|  | Comorbidity > GAS | 0.62 | 0.34 | 0.89 | 0.31 | < .001 |
|  | GAS > UCLA | 1.26 | 1.01 | 1.50 | 0.57 | < .001 |
|  | Direct | 0.95 | 0.46 | 1.44 | 0.22 | < .001 |
|  | Total | 1.72 | 1.15 | 2.30 | 0.39 | < .001 |
| GDS-pos | Indirect | 0.61 | 0.28 | 0.95 | 0.14 | **< .001** |
|  | Direct | 0.16 | 0.08 | 0.24 | 0.27 | < .001 |
|  | Comorbidity > GDP-pos | 3.89 | 3.02 | 4.76 | 0.51 | < .001 |
|  | GDS-pos > UCLA | 1.11 | 0.61 | 1.61 | 0.25 | < .001 |
|  | Total | 1.72 | 1.15 | 2.30 | 0.39 | < .001 |
| CA | Indirect | -0.08 | -0.43 | 0.27 | -0.02 | 0.652 |
|  | Direct | 0.94 | 0.72 | 1.16 | 0.52 | < .001 |
|  | Comorbidity > CA | -0.09 | -0.46 | 0.29 | -0.04 | 0.651 |
|  | CA > UCLA | 1.80 | 1.13 | 2.48 | 0.41 | < .001 |
|  | Total | 1.72 | 1.15 | 2.30 | 0.39 | < .001 |
| IADL | Indirect | 0.38 | 0.08 | 0.69 | 0.09 | **0.014** |
|  | Comorbidity > IADL | -0.31 | -0.40 | -0.22 | -0.46 | < .001 |
|  | IADL > UCLA | -1.24 | -2.17 | -0.31 | -0.19 | 0.009 |
|  | Direct | 1.34 | 0.71 | 1.97 | 0.31 | < .001 |
|  | Total | 1.72 | 1.15 | 2.30 | 0.39 | < .001 |
| ERQ-expressive | Indirect | 0.57 | 0.27 | 0.87 | 0.13 | **< .001** |
|  | Comorbidity > ERQ-expressive | -0.52 | -0.74 | -0.30 | -0.32 | < .001 |
|  | ERQ-expressive > UCLA | -1.10 | -1.43 | -0.76 | -0.41 | < .001 |
|  | Direct | 1.15 | 0.60 | 1.70 | 0.26 | < .001 |
|  | Total | 1.72 | 1.15 | 2.30 | 0.39 | < .001 |

MSPSS-total - Multidimensional Scale of Perceived Social support - Total score; GAS- Geriatric Anxiety scale; GDS-pos - Geriatric Depression Scale - lack of positive mood; CA- the 6-item test indicating cognitive ability; IADL - Lawton-Brody Instrumental Activities of Daily Living Scale; ERQ-expressive- Emotional Regulation Questionnaire - Expressive Suppression
